# Impact of a healthy lifestyle intervention program during pregnancy on women and newborn: STUDY PROTOCOL for a quasi-experimental study

**DOI:** 10.1016/j.pbj.0000000000000029

**Published:** 2018-08-21

**Authors:** Ana Silva, Beatriz Pereira, Sérgio Souza, Carla Moreira, Cláudia Guerra, Cátia Ferreira, Luís Lopes, Rafaela Rosário

**Affiliations:** a School of Education, University of Minho, Braga; b Research Centre in Physical Activity, Health and Leisure, Faculty of Sport, University of Porto, Porto; c Research Centre in Child Studies, Braga; d Senhora da Oliveira Hospital, Guimarães; e School of Nursing, University of Minho, Braga, Portugal

**Keywords:** intervention program, physical activity, pregnancy

## Abstract

Physical activity during pregnancy assumes an important role in the health of both the pregnant and newborn. Given that physical activity tends to decrease throughout this period, it is essential to inform and encourage pregnant women to acquire healthy lifestyles, enabling them to improve their physical and psychological well-being.

This study aims to evaluate the impact of an intervention program on pregnant, newborn, and gestation outcomes, by increasing physical activity levels during this important period of woman's life.

This study will be conducted with 410 pregnant women in the first trimester. Participants will be recruited through the central hospital or community centers. Following consent and baseline data collection, pregnant women who do not have any medical or obstetric contraindication for physical exercise, will be assigned to the control or intervention groups. There will be 3 assessment periods: baseline (time 1—between the 7th and 10th week of gestation), after the intervention (time 2—between the 1st and 3rd day after delivery), and follow-up (time 3—1 month after delivery). The intervention group will have an intervention program, which comprises 2 terms: (1) teachers’ training delivered by researchers and (2) intervention delivered to pregnant women by trained teachers, which consists in 3 weekly classes of physical exercise (45/50 min each). The control group will have the standard care that is usually provided by health professionals.

The research has been approved by the Subcommittee on Ethics for the Life Sciences and Health of the University of Minho (id: SECVS 086/2015) and by the Ethics Committee for Health from the Central Hospital (id: 056/2014). There is a registration in clinical trials.gov, with the reference NCT03045237 (02/2017).

This study has the potential to increase pregnant's physical activity levels and contribute to programs and policies developed to optimize lifestyles during pregnancy and with implications in newborn outcomes.

## Introduction

Experts have not always been favorable in relation to physical activity (PA) during pregnancy. Nonetheless, nowadays engaging in regular PA is considered an important determinant to improve maternal and neonatal outcomes.^1–3^

For a healthy pregnancy, it is recommended to improve healthy lifestyles, namely, daily PA and a balanced diet, essential for the well-being of both pregnant women and newborn. The American College of Obstetricians and Gynecologists^4^ recognizes the benefits of PA and, unless medical or obstetrical contraindications, recommends that all pregnant women are encouraged to be active at least 30 minutes on most of the days. Sedentary women should start with moderate intensity exercise, a minimum of 15 minutes 3 to 4 times a week, then increasing to 30 minutes 5 times a week.^5^

It is recognized that the lifestyle and habits adopted during pregnancy can affect women's health for the rest of life: control of weight gain, maintenance or improvement of physical capacity,^6^ and the conditions of placental irrigation, tone the muscles most affected during pregnancy (such as pelvic muscles, abdominal area, or lower back)^7^ and helps in postpartum recovery.^8^ Regarding the delivery, PA may decrease its duration and the need for epidural,^8^ in addition to a reduction in the number of births by caesarean section.^9^ The increase in exercise level before, during, and after pregnancy contributes positively in the prevention and treatment of several diseases such as pre-eclampsia,^10^ urinary incontinence,^7^ gestational diabetes,^11,12^ low back pain,^13^ and preterm birth.^11^ For symptoms of depression such as mood, insomnia, and anxiety, most studies point toward an improvement of these complaints, in combination with a better general condition and a more energetic and optimistic attitude.^14^

PA also influences the health of the newborn^6^ by improving the Apgar score at first and fifth minutes,^4^ and the intrauterine environment modulations, which will influence the fetal development and consequently child's life.^15^ By helping with control of the gestational weight gain,^16^ it also reduce the risk of macrossomia, chronic conditions, and birth defects.

Despite all benefits described previously, it is well known that the level of PA decreases since the beginning of pregnancy,^17^ whether PA is performed at work and/or leisure.^18^ This fact is confirmed in the study of Currie et al,^19^ which indicates that the moderate and vigorous PA declined the most between the second and third trimester.

Until now, there are few intervention programs developed and implemented during pregnancy. Therefore, we will develop the intervention program “Healthy Bellies,” a partnership between University of Minho, local Hospital, and the City Hall.

The study aims to evaluate the impact of an intervention program, based on the promotion of PA, on pregnant, birth, and newborn outcomes. We hypothesize that the compliance to the intervention program is associated to a reduced (1) gestational weight gain and weight retention postpartum and (2) depressive symptoms. In addition, the intervention program will benefit the delivery (type, duration, complications) and improve infant's birth weight and length.

## Methods

### Recruitment

In the hospital, during the first trimester biochemical screening (between the 7th and 10th week of gestation), 410 pregnant women will be invited to participate in this quasiexperimental study. Participants who want to engage in the PA program will be assigned to the intervention group after the 12th week and the others will be included in the control group, following the standard care procedures provided by health professionals in Portugal (205 women in each group).

### Participants

To be a part of this study, participants will have to accomplish the following criteria: have >18 years old and provide evidence by the medical doctor attesting that there are not any medical or obstetrical contraindications to practice exercise. Exclusion criteria will include pregnant who present absolute or relative contraindications to aerobic exercise during pregnancy according to American College of Obstetricians and Gynecologists guidelines.^4^

It is also required at least the accomplishment of 10 classes so that the participants are included in the intervention group.

### Overview of the program

The intervention program will be based on Bandura theory, which states that the social environment in which people live is part of life and contributes significantly to human development, adaptation, and behavioral changes. People learn and acquire experiences observing the consequences within their environment, as well as the experiences of the people to whom they live. In this context, it is expected a behavioral change resulting from experiences.

The program comprises 2 terms: (1) teachers’ training delivered by researchers and (2) intervention delivered to pregnant women by trained teachers.

During 5 weeks, 6 teachers of Physical Education from the local center of the city hall will have 10 sessions of training (2 h each session). This training workshop, entitled “Healthy lifestyles in pregnancy,” will include the following information: (i) PA during pregnancy (session 1, 2, and 3); (ii) stages of pregnancy, cares, and recommendations (session 4); (iii) nutrition in pregnancy (session 5); (iv) motor development in the first months of life (session 6); and (v) PA strategies in daily living during pregnancy and postpartum (session 7, 8, 9, and 10). In addition, trained teachers will have a meeting once a month during the period of the intervention with the researchers to discuss and reflect about their intervention with pregnant women.

Participants allocated to the intervention group will perform a PA program, 3 times per week, one of which will be developed in the aquatic environment. Classes will last for 45/50 minutes, divided as follows warm up (7/8 min), fundamental part (30 min), and return to calm (10 min). The exercises performed are of moderate/vigorous intensity, and includes aerobic workout, strength, coordination, and flexibility. The program will include every trimester a session about nutrition developed for pregnant.

### Outcome measures

Data will be collected in 3 stages: (1) at the beginning of the program, (2) between first and third day after delivery, and (3) 1 month after delivery.

At baseline (stage 1), information about the sociodemographic profile will be collected, as well as medical history, eating and PA habits, symptoms of depression, and anthropometry. At stage 2, depressive symptoms, PA habits, childbirth information, and anthropometric data of the mother and the newborn will be gathered. At stage 3, depressive symptoms, PA, and food habits of the mother as well as anthropometric data of mother and newborn will be collected.

### Physical activity and sedentary time

PA will be gathered by questionnaire and accelerometry. Participants will use accelerometers GT1M Actigraph for 5 consecutive days (3 weekdays and 2 weekend days). The accelerometer will be attached at the right hip, with the sensor facing up, and the participants will be instructed to use it during waking hours and remove it during sleep and activities involving immersion in water, according to established procedures. In the current study, data will be collected in 2 seconds, each period of 60 minutes of consecutive zeros will be detected as nonwear time and at least 8 hours of data will be considered as a valid day.^20^ The total counts for minute will be converted to light, moderate, and vigorous activity (100–2019 counts/min for light PA, 2020–5998 counts/min for moderate PA and ≥5999 counts/min for vigorous PA) according to the cut points developed by Freedson et al.^21^ Sedentary behavior will be defined as <100 counts/min.^22^

In addition, the questionnaire “Pregnancy Physical Activity Questionnaire” validated for the Portuguese pregnant women^23^ will be used. The Pregnancy Physical Activity Questionnaire consists of 32 questions, grouped into different types of activity: household/caregiving, occupational, sports/exercise, transportation, and inactivity. Participants will estimate the time spent in each activity (none, no <30 min per day, between 30 min and 1 h per day, between 2 and 3 h per day and 3 h or more per day). The activities will be categorized by intensity as sedentary in METs (metabolic equivalent of task) (<1.5 METs), light (1.5–3.0 METs), moderate (3.1–6.0 METs), or vigorous (>6.0 METs). The duration of time spent in each activity is multiplied by its intensity to arrive at a measure of average weekly energy expenditure (MET-h week-1) attributable to each activity.^24^

### Dietary intake

Dietary intake of pregnant women in stages 1 and 3 will be obtained based on food frequency questionnaire, which has also been validated for the Portuguese population.^25^ This is a food list questionnaire in which the participants are asked to estimate the usual frequency of consumption during a specific period of time. Household measures (eg, glasses or tablespoons), food packaging, manufacturer's weight, and energy and nutritional uptake will be estimated using the software Food Processor Plus (ESHA Research Inc, Salem, OR), which includes, along with nutritional information from the United States, typical Portuguese culinary dishes, according to national information of the composition of Portuguese food table.^26^ Also, it will include raw and/or processed foods.

### Socioeconomics

The sociodemographic profile will be evaluated by a questionnaire constructed for this purpose divided into 2 sections. The first will include questions about the subject's sociodemographic characteristics (age, education level, current work status, and socioeconomic status using the Graffar index). The second section will collect general information on lifestyle habits (exercise habits of both pregnant and husband and tobacco consumption) and previous pregnancies (gestational weight gain, occurrence of gestational diabetes or hypertension, and type of birth).

### Anthropometry

We will calculate the body mass index (kg/m^2^) of the mother, through self-reported weight and height, according to the information of “pregnancy book” that all pregnant have in Portugal. Clinical information is recorded in this book, including anthropometry in all months. Through this weight data, we will calculate the body mass index in the baseline (weight before pregnancy), weight gain during pregnancy (the difference between the weight measured at the end of pregnancy and the weight before pregnancy), and the weight loss 1 month after delivery (difference between weight at the end of pregnancy and the weight 1 month after birth).

Weight, length, and cephalic perimeter of the newborn will be collected by analysis of clinical information.

### Childbirth data

These data will be collected in the hospital through the analysis of the clinical information, including Apgar index, admission in neonatal care unit, neonatal morbidity, pregnancy complications (hypertensive disorders, gestational diabetes, preterm delivery, and other complications) gestation age at the delivery, labor onset (spontaneous or induced), labor duration and evolution, way of delivery, third and fourth grade perineal lacerations, epidural analgesia, and episiotomy will also be gathered in the hospital through analysis of clinical process.

### Depressive symptoms

The information related to depressive symptoms will be analyzed by the “Edinburgh Postnatal Depression Scale” questionnaire, previously used in Portugal.^27^ This questionnaire contains 10 questions, each with 4 possible answers and reports to the 7 days preceding the filling.

In Table 1 are presented the instruments used in each stages.

**Table 1:**
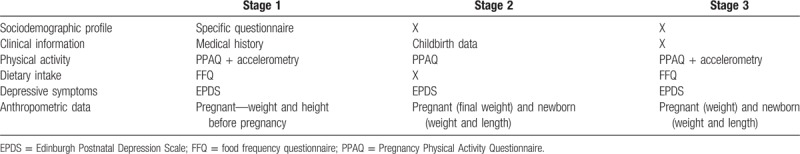
Outcome measures

### Ethics approval and consent to participate

Before starting the program, participants will be informed about the purpose of the study and will provide a written informed consent, according to the ethical standards laid down in the Declaration of Helsinki. In addition, this study was approved by the Subcommittee on Ethics for the Life Sciences and Health of the University of Minho (id: SECVS 086/2015) on October 28, 2015 and by the Ethics Committee for Health of the Central Hospital (id: 056/2014) on December 18, 2015.

### Statistical analysis

Measures of central tendency and dispersion will be calculated for all variables of interest. Comparisons between the baseline and the endpoints of the 2 groups of the study will be performed using multivariate regression models, to address the influence of the PA program in maternal and neonatal outcomes. The data analysis will be performed using IBM SPSS, Version 23.0 (SPSS Inc, Chicago, IL), with a significance level of 0.05.

### Sample size

**Figure 1:**
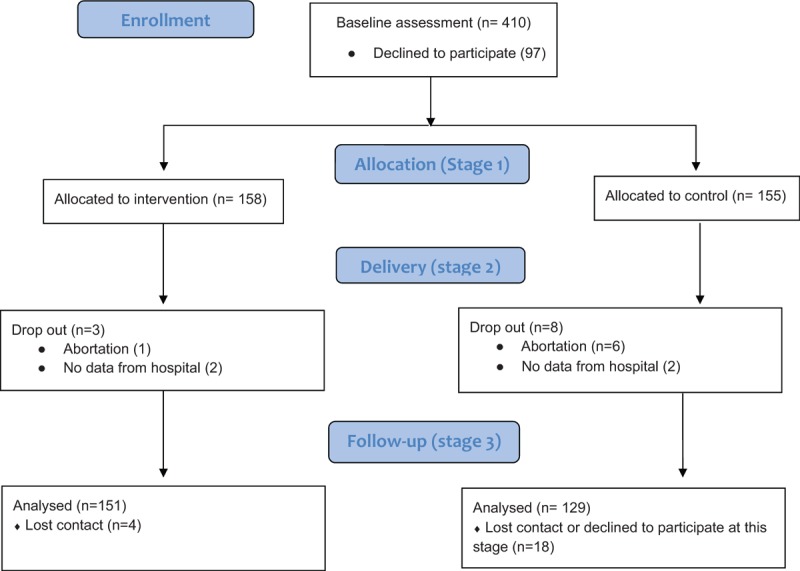
CONSORT 2010 Flow Diagram.

For sample size estimation we will use the variable of interest PA intensity in METs/h/wk. In Portugal, the PA average in pregnant is 210.35 ± 116.75 METs/h/wk.^28^ To detect 20% difference in PA between groups in the third trimester (increasing average PA in intervention group by ≈42 METs/h/wk), with type I and II errors of 5% and 20%, we will have an effect size of 0.36. A sample of 244 pregnant women (122 in each intervention and control group) is anticipated to stage 3. Assuming a dropout of 30%, we propose a sample size by stage 1 of 318 pregnant women, 159 per group; Fig. 1).

## Discussion

Increasing PA during lifespan, including pregnancy, has been strongly associated with and improvement of health and quality of life. This regular practice provides an improvement in the general health of individuals, with relevant results in cardiorespiratory, metabolic, and functional health.^29^ Although the associations between PA during pregnancy and health are well-defined, a cross-sectional study in China with 1056 pregnant women found that only 11% had a compliance to PA international guidelines of 150 minutes a week.^18^ In Portugal, it is known that there is a tendency to decrease PA during pregnancy.^28^

Knowing that interventions targeting excessive weight gain during pregnancy still remain a major challenge^30^ and that some of the pregnancy outcomes investigated showed conflicting findings,^31^ it is important to find the best practices and policies to improve healthy lifestyles in pregnancy and the postpartum period, with implications beyond the improvement of PA, such as in quality of life and newborn outcomes.

Therefore, programs based on PA, accounting for specific characteristics of the population must be developed, guided by a multidisciplinary team of health and educational professionals, in accordance with international guidelines.

We anticipate limitations for the study. First, this is not a randomized controlled trial and we cannot guarantee that participants in the control group do not do any physical exercise and there are also a higher drop out in the control group at stage 3 due to the lack of contact with these participants during the program.

Phone calls and face-to-face meetings will be made to improve compliance to the intervention program and support the participants’ needs and questions that may arise. These contacts will also be fundamental to reduce drop out at follow-up.

## Conclusions

To the best of our knowledge, this is the first intervention program that will be developed in Portugal, contributing with new insights to the topic of lifestyles during pregnancy. In addition, the program will contribute for the development guidelines about PA and sedentary behavior in pregnancy.

In Portugal, PA teachers do not have specific education and training in PA strategies for pregnant women. We expect that the training sessions developed will improve their knowledge and skills regarding PA during pregnancy.

## Acknowledgments

None

## Author contributions

AS drafted the manuscript and all authors have critically evaluated it. AS, BP, SS, and RR substantially contributed to the study design. All authors read and approved the final manuscript. All authors critically reviewed the manuscript, approved the final version, and are fully aware of this publication.

## Conflicts of interest

All authors declare that they have no competing interests.
